# Dental pulp stem cells can improve muscle dysfunction in animal models of Duchenne muscular dystrophy

**DOI:** 10.1186/s13287-020-02099-3

**Published:** 2021-01-25

**Authors:** Yuko Nitahara-Kasahara, Mutsuki Kuraoka, Posadas Herrera Guillermo, Hiromi Hayashita-Kinoh, Yasunobu Maruoka, Aki Nakamura-Takahasi, Koichi Kimura, Shin’ichi Takeda, Takashi Okada

**Affiliations:** 1 Department of Biochemistry and Molecular Biology, Nippon Medical School, Tokyo, Japan; 2 Division of Cell and Gene Therapy, Nippon Medical School, Bunkyo-city, Tokyo, Japan; 3 Department of Molecular Therapy, National Institute of Neuroscience, National Center of Neurology and Psychiatry, Kodaira, Tokyo, Japan; 4 Laboratory of Experimental Animal Science, Nippon Veterinary and Life Science University, Musashino, Tokyo, Japan; 5 Division of Molecular and Medical Genetics, Center for Gene and Cell Therapy, Institute of Medical Science, The University of Tokyo, Minato-city, Tokyo, Japan; 6 Department of Pharmacology, Tokyo Dental College, Tokyo, Japan; 7 Department of General Medicine, The Institute of Medical Science, The University of Tokyo, Minato-city, Tokyo, Japan

**Keywords:** Dental pulp stem cells, Duchenne muscular dystrophy, Anti-inflammatory therapy

## Abstract

**Background:**

Duchenne muscular dystrophy (DMD) is an inherited progressive disorder that causes skeletal and cardiac muscle deterioration with chronic inflammation. Dental pulp stem cells (DPSCs) are attractive candidates for cell-based strategies for DMD because of their immunosuppressive properties. Therefore, we hypothesized that systemic treatment with DPSCs might show therapeutic benefits as an anti-inflammatory therapy.

**Methods:**

To investigate the potential benefits of DPSC transplantation for DMD, we examined disease progression in a DMD animal model, *mdx* mice, by comparing them with different systemic treatment conditions. The DPSC-treated model, a canine X-linked muscular dystrophy model in Japan (CXMD_J_), which has a severe phenotype similar to that of DMD patients, also underwent comprehensive analysis, including histopathological findings, muscle function, and locomotor activity.

**Results:**

We demonstrated a therapeutic strategy for long-term functional recovery in DMD using repeated DPSC administration. DPSC-treated *mdx* mice and CXMD_J_ showed no serious adverse events. MRI findings and muscle histology suggested that DPSC treatment downregulated severe inflammation in DMD muscles and demonstrated a milder phenotype after DPSC treatment. DPSC-treated models showed increased recovery in grip-hand strength and improved tetanic force and home cage activity. Interestingly, maintenance of long-term running capability and stabilized cardiac function was also observed in 1-year-old DPSC-treated CXMD_J_.

**Conclusions:**

We developed a novel strategy for the safe and effective transplantation of DPSCs for DMD recovery, which included repeated systemic injection to regulate inflammation at a young age. This is the first report on the efficacy of a systemic DPSC treatment, from which we can propose that DPSCs may play an important role in delaying the DMD disease phenotype.

## Background

Duchenne muscular dystrophy (DMD) is a progressive and fatal X-linked recessive inherited skeletal and cardiac muscle disorder. It is the most common muscular dystrophy, affecting 1 in 3500 male births [1]. The dystrophin-glycoprotein complex deficiency of the sarcolemma results from mutations in the dystrophin gene and causes progressive degeneration/regeneration cycles in the striated muscle, manifesting as muscle weakness and eventual skeletal muscle atrophy [2, 3]. DMD is a primary degenerative myopathy with a necrotizing phase with secondary inflammation. Consequently, steroids are widely used to improve muscle strength in DMD patients [4–6]. However, the beneficial effects of steroid therapy, including glucocorticoid administration, vary from patient to patient, and administration of these compounds may have side effects. In principle, severe inflammation regulation in muscle tissues can prolong the duration of therapeutic effects.

Multipotent mesenchymal stromal cells (MSCs) express several common cell-surface antigenic markers, such as CD44, CD73, CD90, and CD105, and low levels of major histocompatibility complex class I molecules, without expressing hematopoietic markers CD34 or CD45 [7]. Although originally identified in the bone marrow [7], MSCs can be extracted from numerous tissues including adipose [8], peripheral blood [9], cord blood [10], and amnion [11]. Dental pulp stem cells (DPSCs) obtained from the decidual tooth tissue are a less invasive cell source that shows multipotency [12], as well as high proliferative and immunosuppressive activities [13]. DPSCs can also inhibit the proliferation of phytohemagglutinin-stimulated T cells more strongly than BM-MSCs [14].

The main purpose of DMD treatment is to recover motor function by restoring the expression of dystrophin and to delay pathological progression by suppressing inflammation. Cell-based therapy for DMD has the potential to restore dystrophin expression and restore the muscle parenchyma using hematopoietic stem cells, myoblasts, muscle-derived stem cells, and mesoangioblasts in DMD model mice or Golden retriever muscular dystrophy (GRMD) [15–18]. We have also reported that BM-MSCs could be implanted into the injured muscles of canine X-linked muscular dystrophy in Japan (CXMD_J_), a beagle-based dog colony [19], and successfully used for long-term engraftment in the myogenic lineage [20]. However, improvement of whole-body muscle function and long-term therapeutic effects have not been sufficiently demonstrated by cell therapy.

Our clinical interest in DPSCs for therapeutic cellular applications is based on their anti-inflammatory properties. Therefore, systemic DPSC administration is expected to benefit the immune-modulatory effect in dystrophic muscles and has the capacity to ameliorate progressive DMD. Here, we evaluated the efficacy and safety of systemic DPSC treatment for DMD using animal models. CXMD_J_ shows affected temporalis and limb muscles at a young age, which are comparable to those observed in patients [21–23]. Through experiments using *mdx* mouse and CXMD_J_ models, we demonstrate a therapeutic strategy for long-term functional recovery in DMD using repeated DPSC administration.

## Materials and methods

### Animals

C57BL/6-background *mdx* mice, which show phenotypes similar to C57BL/10 *mdx* mice [24, 25], were a generous gift from Dr. T. Sasaoka (National Institute for Basic Biology). C57BL/6 (WT) mice were purchased from Nihon CLEA (Tokyo, Japan). All experiments using mice were performed in accordance with the guidelines approved by the Nippon Medical School and National Center of Neurology and Psychiatry (NCNP) Animal Ethics Committees. Beagle-based CXMD_J_ colony dogs were bred and housed at NCNP [21]. CXMD_J_ used for cell transplantation and healthy Beagle dogs as controls were cared for and treated in accordance with the guidelines approved by the Ethics Committee for the Treatment of Laboratory Animals at NCNP.

### Cell preparation

Pluripotent stem cell-enriched human dental pulp-derived cells (hDPSCs) were provided by JCR Pharmaceuticals (Hyogo, Japan). The cells were cultured in Dulbecco’s modified Eagle medium (Thermo Fisher Scientific, Waltham, MA) supplemented with 10% fetal bovine serum (Thermo Fisher Scientific) and 1% antibiotic-antimycotic solution (FUJIFILM Wako Pure Chemical Industries, Osaka, Japan) at 37 °C in an atmosphere containing 5% CO_2_.

### Systemic delivery procedure

Systemic delivery of hDPSCs into *mdx* mice was conducted using four injections with an interval of 1 week between injection doses of 1.0 × 10^6^ cells (high dose) or 5.0 × 10^5^ cells (low dose) starting at 4–5 weeks of age (body weight (BW) > 10 g). Age-matched mice were used as controls for the experiments. The experiments using CXMD_J_ were performed using hDPSC administration in the acute phase at 2–3 months of age (Table 1). Polaramine (chlorpheniramine maleate, 0.15 mg/kg, MSD) pretreated CXMD_J_ (three subjects) were intravenously injected with hDPSCs (4.0 × 10^6^ cells/kg/week) at a rate of 1 mL/min. Four injections at weekly intervals were performed as the first course, followed by four injections carried out as the second course after 8–13 weeks. Littermate CXMD_J_ were untreated controls that were injected with saline at identical time intervals.

**Table 1:** Summary of transplantation experiments

Dog ID	Sex	Age^a^	BW^b^	Cell	Cell number	Interval	Injection number
12202MA	M	2	3.2	–	–	–	–
12205MA	M	2	4.1	hDPSCs	4.0 × 10^6^	1 week (1st and 2nd cool)	8
13201MA	M	3	3.6	hDPSCs	4.0 × 10^6^	1 week (1st and 2nd cool)	8
13303MA	M	3	3.8	–	–	–	–
13304MA	M	3	4.0	hDPSCs	4.0 × 10^6^	1 week (1st and 2nd cool)	8
14102MA	M	3	3.3	–	–	–	–

*M* male

^a^Age at injection (months)

^b^*BW* body weight at first injection (kg)

### Biodistribution of hDPSCs

DNA extractions were performed on tissue suspensions using a DNeasy Blood and Tissue kit (Qiagen, Valencia, CA) and quantified using a spectrophotometer (NanoDrop; Thermos Fisher Scientific). Real-time quantitative PCR was performed using the DNA Master SYBR Green I kit (Roche Diagnostics, Basel, Switzerland) and primers for the human *Alu* site. The primer sequences used were as follows: 5′-GTCAGGAGATCGAGACCATCCC-3′ (forward) and 5′-TCCTGCCTCAGCCTCCCAAG-3′ (reverse). PCR conditions were as follows: 95 °C for 2 min, followed by 40 cycles at 95 °C for 15 s, 68 °C for 30 s, and then 72 °C for 30 s. Standards were generated by adding 10-fold serial dilutions of hDPSCs to determine the concentration of hDPSCs in genomic DNA.

### Blood tests

Hematological and serum biochemical testing were performed using a semiautomatic hematology analyzer (Sysmex Hematology Analyzer F-820; Sysmex, Hyogo, Japan). Serum alkaline phosphatase (ALP), aspartate transferase (AST), and blood urea nitrogen (BUN) levels were measured using an automated analyzer (DRI-CHEM3506; Fuji Film Medical, Tokyo, Japan). C-reactive protein (CRP) levels were measured using a colorimetric assay with an FDC3500 clinical biochemistry analyzer.

### ELISA

The serum IL-6 levels were determined using a Quantikine ELISA mouse kit (R&D Systems, Minneapolis, MN). A canine IL-6 immunoassay (R&D Systems) was carried out according to the manufacturer’s recommendations.

### Grip strength

Forelimb grip strength was measured using a grip strength meter (MK-380 M; Muromachi Kikai Co., Ltd., Tokyo, Japan) as previously described [26]. Five trials were performed with a resting period of 5 s between trials. The average tension force (g) was calculated from 3 highest measurements for each group of mice.

### Analysis of locomotor activity

Physiological mouse activity was analyzed in each cage with a computerized wheel system (dual activity monitor system, SHINFACTORY Co., Ltd., Fukuoka, Japan) by counting the number of wheel revolutions during each 5 min interval using ACTIMO-DATA II software [27]. The activity of dogs was monitored and counted using an infrared sensor system (Supermex, Muromachi Kikai) as previously described [28]. The average daily locomotor activity shown by the dogs over 5 days and nights (12 h light/dark cycles) was calculated. We also compared the 15-m running time of CXMD_J_ during the experimental period. The running speed was averaged from four measurements. To determine the acceleration parameter, we used portable wireless hybrid sensors (TSND121; ATR-Promotions Inc., Kyoto, Japan) on the thoracic and lumbar regions of the dogs, as described previously [29]. The acceleration magnitude (*AM*) was calculated from the three acceleration vectors (*Ax, Ay, Az*) as the square root of the sum of the three-axial values (*AM* = √*Ax*^2^ + *Ay*^2^ + *Az*^2^) [30] and was averaged for each trial. The relative components of the *AM* along the three axes (%) were calculated by dividing the absolute values of each axis by the AM [31], and these components that were averaged in each trial were calculated as acceleration ratios (*Ax* ratio, *Ay* ratio, *Az* ratio).

### Magnetic resonance imaging

Images of the T2-weighted and fat-saturated T2-weighted series were obtained in CXMD_J_ anesthetized animals with an inhalational mixture of 2–3% isofluorane and oxygen according to a method described previously [32] with constant monitoring of heart rate and oxygen saturation. We examined the crus muscles of the lower limbs using a superconducting 3.0-Tesla MRI device (MAGNETOM Trio; Siemens Medical Solutions, Erlanger, Germany) with an 18-cm-diameter/18-cm-length human extremity coil. Quantitative analysis of the images was performed using the Syngo MR2004A software (Siemens Medical Solutions), as previously reported [29, 32]. Briefly, regions of interest (ROIs) were selected to avoid flow artifacts and large vessels. Signal intensities were measured for these ROIs. Signal-to-noise ratios (SNRs) of each ROI were calculated using the following equation: SNR = signal intensity/SDair, where SDair is the standard deviation (SD) of the background noise. The average SNR (Ave SNR) was calculated using the equation described in our previous report [29].

### Hindlimb extensor strength test

The two hindlimbs in CXMD_J_ were evaluated by measuring the wrist flexion and extension strength using a custom-made torque measurement device. Stimulation frequencies of 60 Hz activate muscles that extend or push the hind paw against the ground. A transducer captures the torque generated when the paw pushes against the force plate. The maximal torque was expressed as a percentage of the predicted values computed using a model based on control values [33].

### Histopathology and immunohistochemistry

Transverse cryosections (10-μm thick) prepared from frozen muscle tissues were stained with hematoxylin and eosin (H&E) using standard procedures. Cryosections fixed with 1% paraformaldehyde were treated with anti-canine developmental myosin heavy chain (dMyHC) antibody (NCL-MHCd; Leica) followed by Alexa 568-conjugated anti-mouse IgG antibodies (Thermo Fisher) or canine IgG antibody conjugated with Alexa 488 (Thermo Fisher) as the secondary antibody and mounted in Vectashield (Vector Laboratories Inc., Burlingame, CA) with 4, 6-diamidino-2-phenylindole. Immunofluorescence and H&E staining were visualized using an IX71 and IX81 fluorescence microscope (Olympus, Tokyo, Japan). Quantitative analysis of the myofiber area was performed using cellSence software (Olympus) using H&E images (2500–3500 fibers in each group).

### Echocardiography

Echocardiographic images of unanesthetized dogs were obtained using a Vivid S6 Dimensions (GE Healthcare Japan, Tokyo, Japan) probe equipped with a linear array ultrasound transducer (i13L) transmitting at 10 MHz as described previously [34]. The ejection fraction (EF) (%) was calculated using M-mode parameters based on multiple measurements.

### Statistical analysis

Data are presented as mean ± S.D. Differences between two groups were assessed using unpaired two-tailed *t* tests. Multiple comparisons between three or more groups were performed using one-way or two-way ANOVA. Statistical significance is defined as ^*^P < 0.05, ^**^P < 0.01, ^***^P < 0.001, and ^****^P < 0.0001. Statistical significance was calculated using Excel (Microsoft) and GraphPad Prism 8.

## Results

### Systemic injection of human DPSCs (hDPSCs) into dystrophic mice

**Fig. 1:**
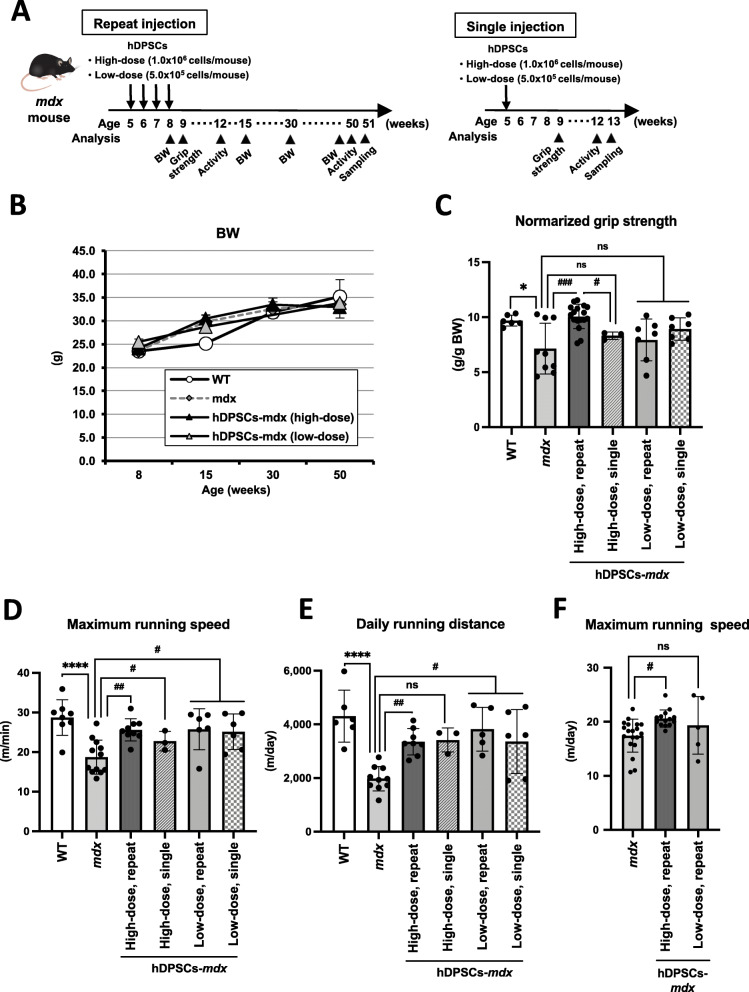
Safety and functional recovery on hDPSC-treated *mdx* mice. **a** Schematic representation of the repeat or single hDPSC-treatment schedule in *mdx* mice. **b** Growth curve of 9 to 30-week-old wild type (WT), *mdx,* and hDPSC-treated *mdx* mice (*n* = 4 each). **c** Normalized grip strength (g/g body weight, BW) measured in 9-week-old WT (*n* = 6), untreated *mdx* (*n* = 9)*,* repeat (*n* = 17, 7), and single (*n* = 3, 7) hDPSC (high- or low-dose)-treated *mdx* mice. **d** Quantification of maximum running speed (m/min) in the wheel cage, and **e** daily distance covered during wheel running in 12-week-old WT (*n* = 6–8), untreated *mdx* (*n* = 10–12)*,* repeat (*n* = 8–9, 5–6), and single (*n* = 3, 6) hDPSC (high- or low-dose)-treated *mdx* mice. **f** Quantification of maximum running speed (m/min) in the wheel cage in 50-week-old *mdx* (*n* = 16–19)*,* repeat hDPSC (high- or low-dose)-treated *mdx* mice (*n* = 13–14, 5). All data are represented as mean ± SD and statistical differences compared to WT (^*^P < 0.05, ^****^P < 0.0001) and untreated *mdx* (^#^*P* < 0.05, ^##^*P* < 0.01, and ^###^*P* < 0.0005), one-way ANOVA

*Mdx* mice received a single dose of hDPSCs (high dose, 1.0 × 10^6^ cells, or low dose, 5.0 × 10^5^ cells) or repeated administration of high- and low-dose hDPSCs via the tail vein (Fig. 1a). None of the hDPSC-treated *mdx* mice showed any significant effect on body weight (BW) during the experiments (Fig. 1b). Grip strength in *mdx* mice showed significant restoration after repeated administration of high-dose hDPSC (Fig. 1c, Table S1, *mdx* vs. repeated high dose of hDPSC-*mdx*, *P* = 0.0002; WT vs. repeated high dose of hDPSC-*mdx*, *P* = 0.995). However, grip strength did not improve in mice administered a single high-dose or repeat low-dose injections. The grip strength of high-dose hDPSC-treated *mdx* mice was not significantly different from that of 1-year-old *mdx* mice or WT mice (Figure S1A).

We examined the progressive resistance during wheel running in *mdx* mice. The hDPSCs-treated *mdx* mice had improved running speed compared to *mdx* mice (Fig. 1d, Table S2, *mdx* vs. repeated high dose of hDPSC-*mdx*, *P* = 0.007; WT vs. repeated high dose of hDPSC-*mdx*, *P* = 0.642; *mdx* vs. single high dose of hDPSC-*mdx*, *P* = 0.664; *mdx* vs. low dose of hDPSC-*mdx*, *P* = 0.019) and had a daily running distance similar to WT mice (Fig. 1e, Table S2, *mdx* vs. repeated high dose of hDPSC-*mdx*, *P* = 0.0069; WT vs. high dose of hDPSC-*mdx*, *P* = 0.214; *mdx* vs. single high dose of hDPSC-*mdx*, *P* = 0.07; *mdx* vs. low dose of hDPSC-*mdx*, *P* = 0.015). Surprisingly, there was a difference in running speed between the repeated treatment and untreated groups at 1 year of age (Fig. 1f, *mdx* vs. repeated high dose of hDPSC-*mdx*, *P* = 0.022), although their daily running distance was not significantly different (Figure S1B and Table S2, *P* = 0.24).

**Fig. 2:**
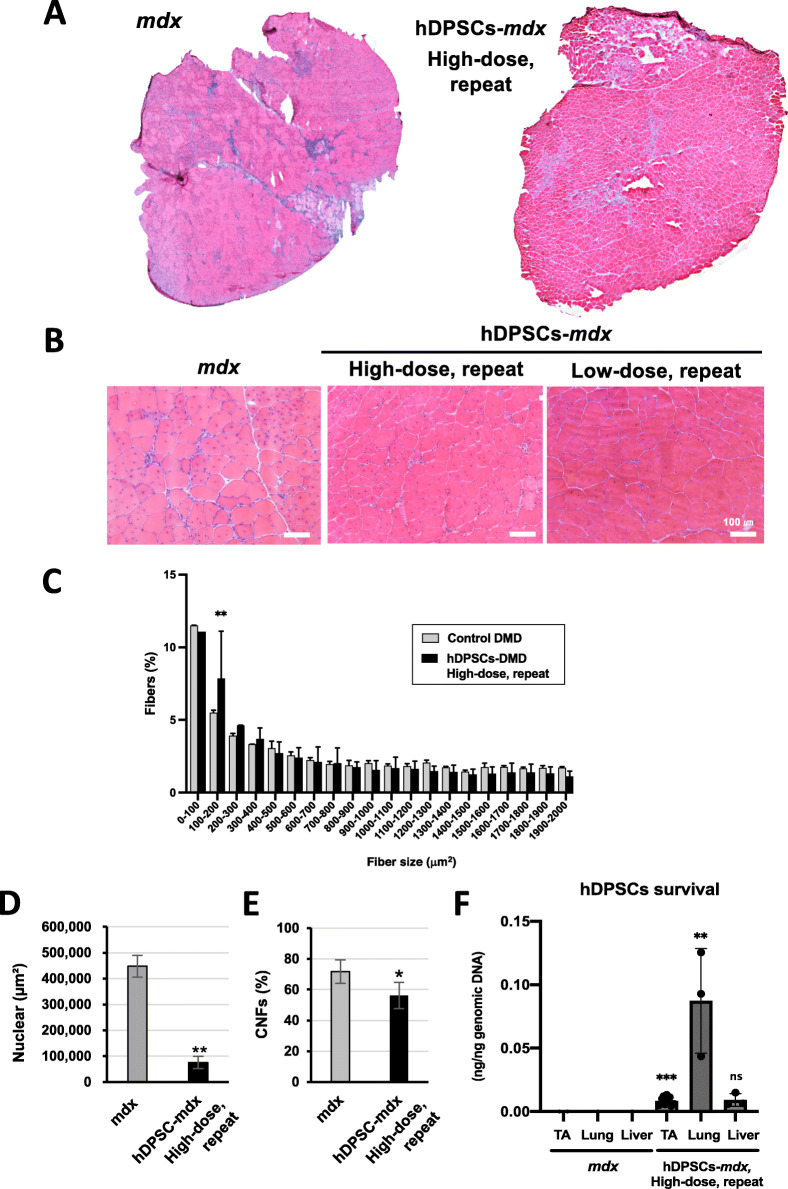
Histopathological appearance of hDPSC-treated *mdx* mice. Hematoxylin and eosin (H&E) staining of overall cross-sections of the tibialis anterior (TA) muscle from 12-week-old untreated *mdx* and high-dose hDPSC-treated *mdx* mice (**a**) and high magnification images containing low-dose hDPSC-treated *mdx* mice (**b**). Scale bars, 100 μm. **c** The average percentage of the frequency distribution of the myofiber area (μm^2^). Area values showed both frequency (% of total fibers) and distribution comparisons, paired *t* test. **d** Quantification of nuclear expansion as cross-section area (*n* = 3) and (**e**) quantification of the percentage of centrally nucleated fibers (CNFs) in the TA muscle (*n* = 4). Statistical differences compared to *mdx* mice (^*^P < 0.05, and ^**^P < 0.01), paired *t* test. **f** Biodistribution of hDPSCs measured by *Alu*-PCR in the TA muscle, lung, and liver tissue from untreated *mdx* and repeated high-dose hDPSC-*mdx* mice 1 week after transplantation. Statistical differences compared to *mdx* mice (^**^P < 0.01, and ^***^P < 0.0005); ns, not significant, two-way ANOVA

The cross-section of the tibialis anterior (TA) muscle of *mdx* mice showed smaller (regenerating fibers) and larger (hypertrophic fibers) fiber diameter in the dystrophic muscles, centrally nucleated fibers (CNFs), spread muscle interstitium, and cell infiltration interspersed in the muscle interstitium (Fig. 2a–d). The histopathological findings observed in the repeatedly hDPSC-treated *mdx* mice included limited muscle interstitium, nuclear infiltration (Fig. 2a, b, d), and reduced frequency of larger fiber areas (Fig. 2c), but not in a dose-dependent manner. We also observed that the CNFs in dystrophic muscle, which are indicative of regenerated myofibers following degeneration, were reduced in the hDPSC-treated *mdx* mice with a repeated high dose (Fig. 2e), suggesting that degeneration was regulated in the hDPSC-treated muscle. When we examined the distribution of hPDSCs by human-specific *Alu*-PCR, 1 week after the transplantation, many cells accumulated in the lung, and some survived in the skeletal muscle (Fig. 2f), but these were detected for only a short period of time.

Altogether, our data supported the conclusion that short-term amelioration of the DMD phenotype was observed in all groups of hDPSC-treated *mdx* mice. Among them, the mice repeatedly treated with high-dose hDPSCs showed long-term and remarkable beneficial effects on the DMD phenotype.

### Safe systemic transplantation of hDPSCs into CXMD_J_

**Fig. 3:**
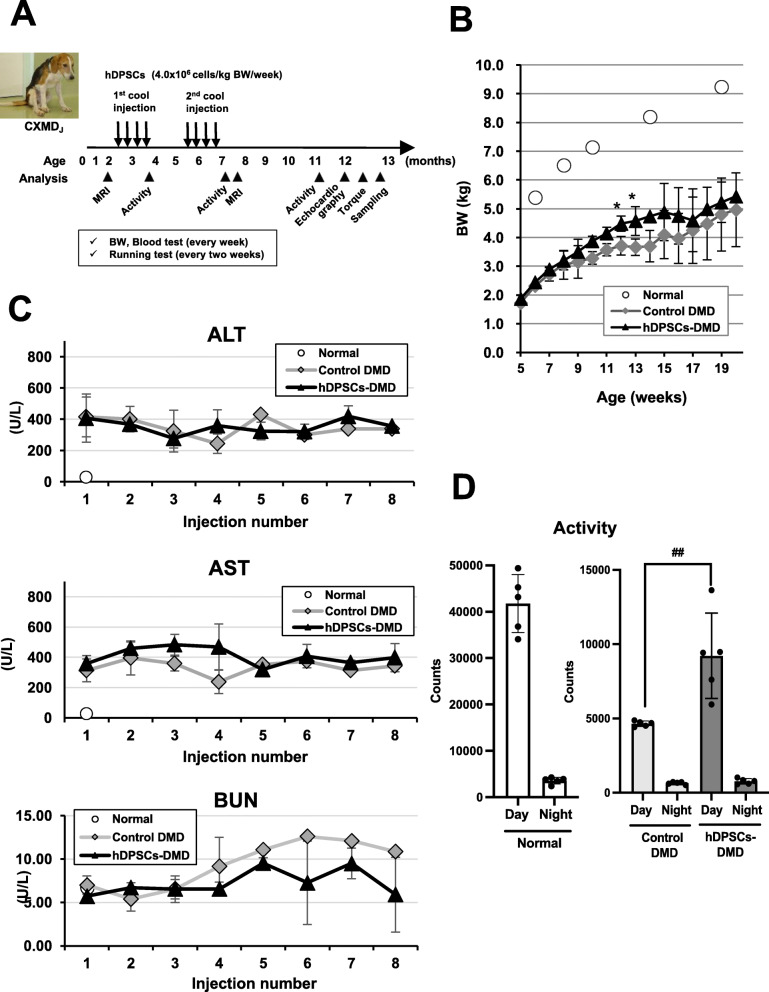
Evaluation of safety and efficacy after repeated systemic administration of hDPSCs into CXMD_J_. **a** Schematic representation of the hDPSC-treatment schedule in CXMD_J_. **b** Early life growth and **c** serum chemistry data, such as alkaline phosphatase (ALP), aspartate aminotransferase (AST), and blood urea nitrogen (BUN) of normal dogs, untreated CXMD_J_ (control DMD; 12202MA, 13303MA, 14102MA), and hDPSC-treated CXMD_J_ (hDPSCs-DMD; 12205MA, 13201MA, 13304MA) after each administration throughout the experimental period. **d** Behavioral activity (day and night) in the home cage of normal dogs (13301MN, 14103MN) and CXMD_J_ (control DMD; 13303MA, 14102MA, and hDPSCs-DMD; 13201MA, 13304MA) at the age of 48–50 weeks, represented as mean activity counts (mean ± SD) observed every day and night. Statistical differences show normal vs. hDPSCs-DMD (^*^P < 0.05), and control DMD vs. hDPSCs-DMD (^##^*P* < 0.01), *t* test

We next investigated the possibility of long-term benefits in hDPSC-treated animals using dog models. We started with the administration of hDPSCs in CXMD_J_ with the DMD phenotype in the acute phase, when the disease signs were already observable (*n* = 3 per group, Fig. 3a, Table 1). Eight systemic injections of 4 × 10^6^ cells/kg were performed on three CXMD_J_ dogs (12205MA, 13201MA, 13304MA) with two courses of weekly injections for 4 weeks (Table 1). After each injection, we carefully monitored the activity, heart rate, respiratory rate, and appearance of any abnormal signs. During development, hDPSC-treated CXMD_J_ showed good growth and no severe weight loss due to continuous administration (Fig. 3b). No obvious abnormalities related to hepato-renal damage or anemia due to systemic administration in all hDPSC-treated CXMD_J_ were noted in blood tests, which included the determination of ALP, AST, BUN levels, and CRP levels (Fig. 3c, Figure S2A).

Spontaneous locomotor activity measured using an infrared sensor system showed a largely reduced mobility of CXMD_J_ with aging [28]. In contrast, hDPSC-treated CXMD_J_ maintained activity for longer periods compared with untreated dogs until they turned 1-year-old (Fig. 3d), suggesting that no serious adverse effects in hDPSC-treated CXMD_J_.

### Regulatory effects of hDPSC treatment on inflammation in CXMD_J_

**Fig. 4:**
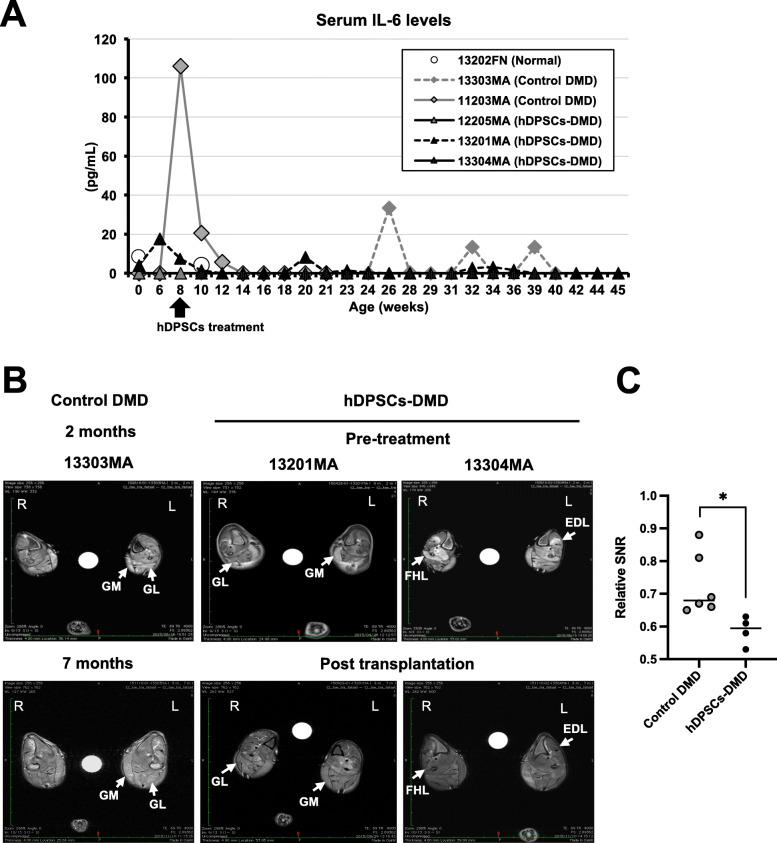
Regulation of inflammation associated with the DMD phenotype. **a** Serum levels of IL-6 from dogs quantified using ELISA during the experimental period. **b** Cross-sectional magnetic resonance images (MRI) in the lower leg muscles of untreated (control DMD) and hDPSC-treated CXMD_J_ (hDPSC-DMD). T2-weighted imaging was comparable in the lower legs (R, right side; L, left side, left/right asymmetry) of CXMD_J_ (untreated 13303MA and hDPSC-treated 13201MA and 13304MA). **c** Quantitative changes of higher T2-signals (signal-to-noise ratio, SNR) in the hindlimb muscles on CXMD_J_ shown in MRI data (**b**). Relative SNR was calculated from the highest signals in each hindlimb of 2-month-old compared to 7-month-old dogs. Data are represented as mean ± SD and statistical differences compared to control DMD (^*^P < 0.05), *t* test

During the experiments, serum IL-6 and TNF-α levels in CXMD_J_ did not increase over the normal range after hDPSC-treatment, whereas an increase was transiently detected in the untreated CXMD_J_ (Fig. 4a, Figure S2B). To address the regulation of progressive inflammation, the intensity of T2-signals on MRI was measured, which is characteristic of necrosis/edema and inflammatory lesions in CXMD_J_. When comparing the quantitative changes of higher T2-signals (4–6 sites) in hindlimb muscles between 2 and 7 months of age, these signals were significantly reduced in the hDPSC-treated CXMD_J_ (Fig. 4b, c, [13201MA, 13303MA], Figure S3 [12205MA], and Table S3). These findings indicate that hDPSC treatment can enhance the regulation of inflammatory lesions in dystrophic muscles.

### Structural stability of the skeletal muscle of hDPSC-treated CXMD_J_

**Fig. 5:**
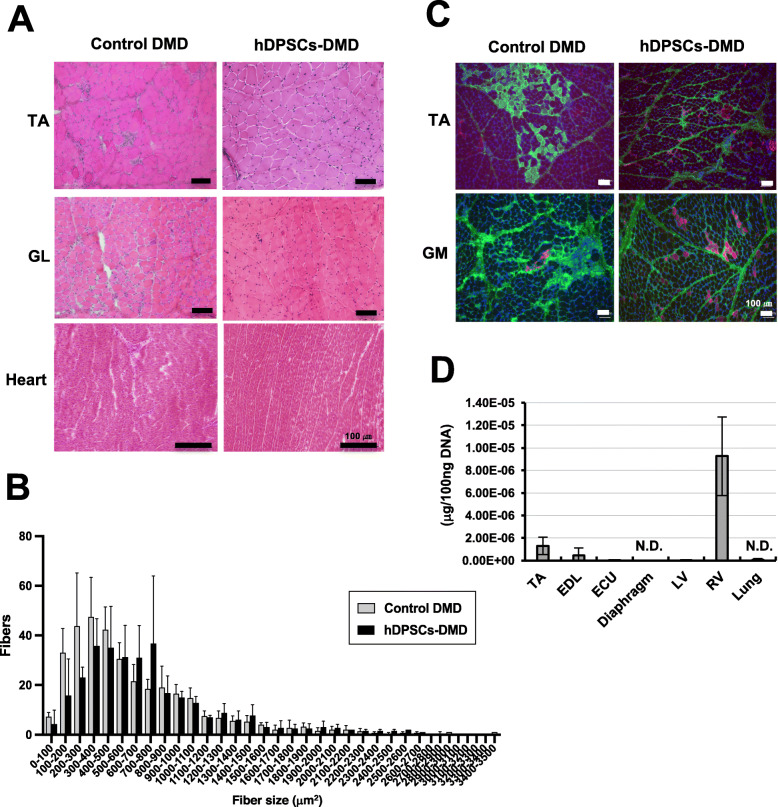
Histopathological examination of hDPSC-treated CXMD_J_. **a** Hematoxylin and eosin (H&E) staining of the tibialis anterior (TA) and the gastrocnemius lateral (GL) muscle, and heart (left ventricular) from 1-year-old control DMD (12202MA, 13303MA, and 14102MA) and hDPSCs-DMD dogs (12205MA and 13304MA). Scale bar, 100 μm. **b** Muscle fiber areas (μm^2^) measured from the TA muscle of control and hDPSC-DMD dogs by H&E staining (*n* = 4). The average number of myofibers is described as the distribution. **c** Immunofluorescence analysis of the TA and gastrocnemius medialis (GM) muscles from the control DMD (12202MA, 14102MA, and 3903MA) and hDPSCs-DMD (12205MA and 13304MA) with anti-IgG antibodies (green) and anti-developmental myosin heavy chain antibodies (red) and DAPI (blue) staining. **d** Biodistribution of hDPSCs measured by *Alu*-PCR in the TA, extensor digitorum longus (EDL), extensor carpi ulnaris (ECU) muscle, diaphragm, heart (left ventricular, LV; right ventricular, RV), and lung tissue from repeated hDPSC-CXMD_J_, 7 weeks after transplantation. N.D., not detected

To investigate the pathological changes in hDPSC-treated muscle, we examined cross-sections of DMD muscles. Dystrophic phenotypes, including nuclear infiltration and spread of muscle fiber interstitium, were downregulated in the skeletal muscle of hDPSC-treated CXMD_J_ (Fig. 5a, Figure S4). Although dystrophic muscles also displayed a high myofiber size variability due to a higher number of smaller fibers and the occurrence of hypertrophic fibers, the fiber size distribution in the TA muscles shifted toward a lower number of both smaller and hypertrophic fibers in the case of hDPSC-DMD (Fig. 5b, *P* = 0.0425, Figure S5). Immunostaining analysis showed significantly decreased accumulation of IgG, a marker for damaged myofiber [32], in the skeletal muscle of hDPSC-treated CXMD_J_ (Fig. 5c). Although the muscle tissue from CXMD_J_ also showed a number of developmental myosin heavy chain (dMyHC)-positive fibers, which are not usually observed in the muscles of normal dogs [35], there were dMyHC-positive fibers only in limited areas within the tissue of hDPSC-treated CXMD_J_, suggesting that systemic hDPSC treatment can improve the dystrophic phenotype. By *Alu*-PCR analysis, we also confirmed that circulating transplanted hDPSCs were not detectable in blood within 48 h after injection (Figure S2C). Seven weeks after treatment, the retention of hDPSCs was confirmed in parts of the skeletal muscle, such as the TA and extensor digitorum longus muscle and cardiac muscle (left ventricular, LV), but not detectable in the lung and diaphragm of recipient dogs (Fig. 5d).

### Improved locomotor activity in hDPSC-treated CXMD_J_

**Fig. 6:**
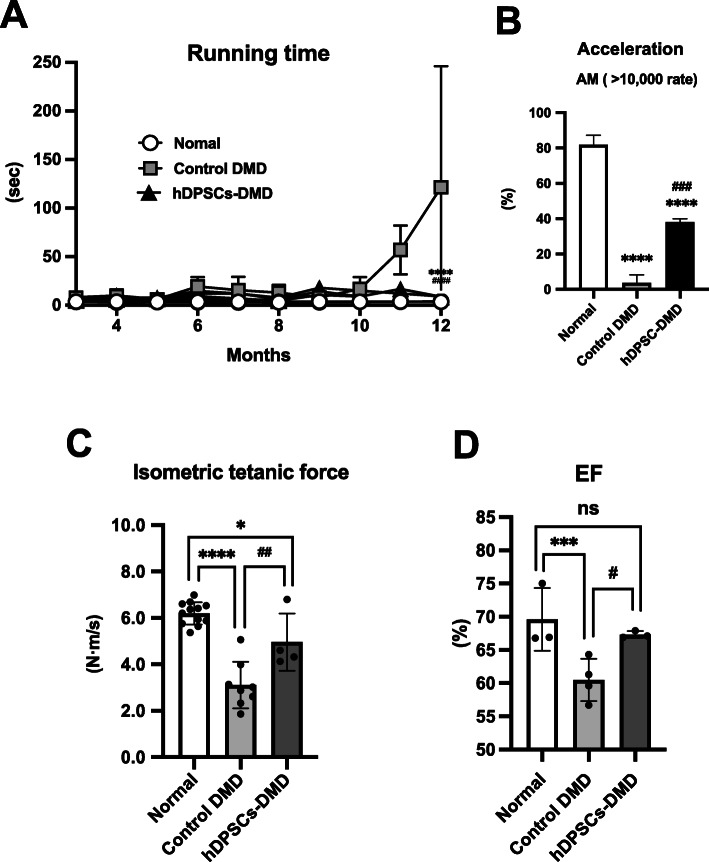
Improvement of muscle function in hDPSC-treated CXMD_J_. **a** Monitoring of 15 m running speed (s) during the experimental period. **b** As an acceleration parameter, the acceleration magnitude (*AM*) was calculated from the three acceleration vectors measured by 15 m running. Hardly detectable ratio of higher *AM* value (> 10,000 mG) was obtained, which was calculated by dividing the total number of *AM* from the number of higher *AM* value (> 10,000 mG) in all groups. **c** Tetanic force estimated on hindlimbs of dogs. The graph shows the summary statistics of force change relative to baseline values 5 weeks after the final injection. **d** Ejection fraction (EF) values were calculated using echocardiographic parameters post-transplantation (normal dogs, 14103MN and 12201MN; control DMD, 13303MA, 13802MA, and 14102MA; and hDPSCs-DMD, 12205MA, 13201MA, and 13304MA). All data are represented as mean ± SD and statistical differences between normal and control DMD dogs (^*^P < 0.05, ^**^P < 0.01, ^***^P < 0.001, and ^****^P < 0.0001), control DMD and hDPSCs-DMD (^#^*P <* 0.05, ^##^*P <* 0.01, ^###^*P *< 0.001, and ^####^*P <* 0.0001), multiple *t* test and one-way ANOVA

The CXMD_J_ model displays progressive clinical impairment with a rapid decline in the walking ability of dogs with progressive weakness, abnormal stiff limbs, and short strides [21, 29]. hDPSC-treated CXMD_J_ showed continued stabilization of clinical status characterized by a higher clinical score maintained up to the age of 12 months (Figure S6), reflecting reduced fatigability, decreased limb stiffness intensity, and less severe ankyloses [36], as described in our previous reports [21, 28, 29, 37]. Indeed, the home cage physiological activity of hDPSC-CXMD_J_ during the daytime (9213 ± 2871 counts) was higher than that of control DMD (4645 ± 183.9 counts, *P* = 0.0075) in 12-month-old dogs (Fig. 3d), even though it was still significantly different from that of normal dogs (41,746 ± 6241 counts, *P* < 0.0001). Video data showed an increased mobility of hDPSC-CXMD_J_ compared to untreated CXMD_J_ in the cage based on jumping and playfulness (Supporting Information, movie S1). These observations encouraged us to investigate whether hDPSC could increase the locomotor activity of CXMD_J_. We monitored the 15 m running speed of CXMD_J_ to determine motor function and confirmed that CXMD_J_ had a slower speed according to their progressive phenotype (Fig. 6a, Supporting Information, movie S2, 3). Meanwhile, hDPSC-treated CXMD_J_ maintained their running speed and were active for more than 12 months (vs. control DMD, *P* < 0.00001; vs. normal, *P* < 0.0005). We also measured multiple acceleration parameters, which severely decrease with age in dystrophic dogs compared to normal dogs, as we have previously reported [29]. When using the acceleration parameter to evaluate motor function, acceleration magnitudes (*AM*) were not significantly different between untreated and hDPSC-treated CXMD_J_ in either the thoracic or lumbar region (Figure S7 and Table S4). Interestingly, the higher *AM* (> 10,000 mG) maintenance ratio was rarely reached in CXMD_J_, but was observed more frequently in hDPSC-treated CXMD_J_ (Fig. 6b).

### Improvement in skeletal muscle and cardiac dysfunction

Finally, we investigated whether repeated systemic administration of hDPSCs would lead to long-term improvement of dystrophic muscle function. An instantaneous force by torque evaluation was used to assess skeletal muscle function. The tetanic force on the CXMD_J_ hindlimbs was 51.0 ± 12.3% (3.12 ± 1.0 N m/s; *P <* 0.0001) compared to normal dogs (6.12 ± 0.49 N m/s), while all hDPSC-treated CXMD_J_ (4.96 ± 1.24 N m/s) showed significantly stronger torque values (81.0 ± 12.8% of normal dogs, *P* = 0.042) compared to untreated CXMD_J_ (*P* = 0.0039) as described in Fig. 6b. With regard to cardiac function, CXMD_J_ shows progressive cardiac dysfunction, which is similar to DMD patients presenting with dilated cardiomyopathy [22, 38]. Echocardiography showed that LV function was maintained, with higher levels of EF in hDPSC-treated CXMD_J_ (mean ± SD, 67.3 ± 0.53%) than that in control DMD (60.5 ± 3.2%, *P* = 0.001), and comparable to that in normal dogs (69.6 ± 4.7%, Fig. 6c).

Altogether, these observations consistently indicate that repeated systemic hDPSC treatment in DMD animals can improve the dystrophic phenotype by maintaining muscle function.

## Discussion

Here, we investigated and proposed a protocol for safe and effective stem cell transplantation aimed at the functional recovery of skeletal muscles. A comprehensive analysis was performed during experiments performed by administering hDPSCs in animal models of DMD. Results from both *mdx* mice and CXMD_J_ models showed that disease progression slowed down after repeated rounds of hDPSC treatment to induce long-term effects.

Short-term amelioration of locomotor function was also observed in all groups of hDPSC-treated *mdx* mice. However, the dose-dependent effects of hDPSC on physiological activity, grip strength, running speed, and longer running distances suggest that sufficient amelioration would require repeated high-dose administration of cells (Fig. 1). The maintained running ability up to 1 year of age in mice repeatedly treated with high-dose hDPSCs in the acute DMD phase also supports the conclusion that the effects are long-term. Since transplanted cells are temporary, but accumulate in muscle tissues, it is generally considered that hDPSCs play a protective role against inflammation in the dystrophic muscle. In our study, this was supported by the histopathological appearance of the hDPSC-treated muscle, with findings such as reduced areas of nucleic infiltration (Fig. 2).

In our experiments using dog models, repeated systemic hDPSC injections into the CXMD_J_ were safe and caused no severe side effects without the need for immunosuppression (Fig. 3). Since hDPSCs share characteristics with clinically used BM-MSCs that lack HLA-DR expression, these cells are not likely to be subjected to immunological attack in the recipient body.

The repeated use of hDPSCs in the CXMD_J_ prevented severe inflammation with an IL-6 and TNF-α surge, as validated by cell infiltration that was much more localized, and attenuation of T2 signals in muscles on MRI (Fig. 4, Figure S2B). Higher concentrations of circulating IL-6, IL-1, and TNF-α have also been reported in DMD compared to that in healthy subjects [39]. These facts indicate that hDPSCs have an immune-modulatory effect in DMD and may attenuate the histopathological changes that lead to dysfunction in dystrophic muscles. Histopathological appearance improvements after hDPSC administration indicate the functional recovery of dystrophic muscle (Fig. 5). Importantly, the home cage activity and running function of hDPSC-treated CXMD_J_ were maintained until they reached 1 year of age (Figs. 3d and 6a). The therapeutic effects of hDPSCs are considered to be more effective in the long-term maintenance of running function, a capacity that diminishes with age in the disease, rather than contributing to recovery. It appears that hDPSC treatment at a young age could alleviate the DMD phenotype by preserving the whole-body muscle function.

Our results included an acceleration parameter to determine the instantaneous running ability of CXMD_J_ (Fig. 6b). Since there is a large difference in the evaluation of running speeds among individuals, and there are issues for some DMD patients in walking for even for 6 min, introducing the acceleration parameter into the evaluation of running ability could be applied to assess outcomes in clinical trials for hereditary neuromuscular disorders, including DMD.

Significantly stronger isometric torque values in hDPSC-treated CXMD_J_ clearly demonstrate that the progressive loss in limb muscle strength is ameliorated by repeated hDPSC treatment (Fig. 6c). Echocardiography showed that decreased EF in CXMD_J_ due to progressive cardiac dysfunction [22, 38] was rescued in the hDPSC-treated CXMD_J_ (Fig. 6d), suggesting that hDPSC treatment improved not only limb muscle strength but also cardiac muscle function. Since DMD patients sometimes exhibit dilated cardiomyopathy, the DPSC therapies presented in this study may be promising for maintaining cardiac function.

It has also been reported that transplantation of hDPSCs in the GRMD model improved muscle pathology, resulting in limited dystrophin expression [40]. Since hDPSCs can differentiate into the myogenic lineage only with very low efficiency without the use of agents, including the demethylating agent 5-aza-2-deoxycytidine [41], our study indicates that the benefits obtained from hDPSCs depend on their function as anti-inflammatory agents, and not by direct contribution to tissue repair. We examined the possibility of dystrophin expression derived from hDPSCs in the skeletal muscle but did not confirm the presence of dystrophin mRNA by reverse transcription PCR (Figure S8). In fact, the restoration of dystrophin protein levels is the major target for the treatment of DMD patients. In contrast, the benefit of DPSCs in this study is likely dependent on their role as a systemic anti-inflammatory agent and not their differentiation directly promoting muscle fiber regeneration. The present innovation is a therapeutic approach utilizing the inflammation-regulating ability of MSCs. Since stem cells other than MSCs do not exhibit such ability, this function can be expected to be novel.

We previously provided evidence that severe phenotypes in IL-10 knockout *mdx* mice, such as increased M1-macrophage infiltration, high inflammatory factor levels, and progressive cardiorespiratory dysfunction, show a predisposition toward inflammation [42]. Glucocorticoids are widely used in patients to interrupt and improve muscle strength during early stages, which may also act directly on muscle fibers by stabilizing the sarcolemma [43, 44]. However, this is frequently associated with severe side effects. Several anti-inflammatory therapies reportedly have beneficial effects on DMD phenotypes [45, 46]. TNF-α blockers, such as infliximab, have been investigated using *mdx* mice as an anti-inflammatory agent for DMD [45, 47]. Proteasome inhibitors, such as bortezomib, have been shown to block NF-*κ*B activation, improve the appearance of muscle fibers, and reduce both connective tissue deposition and inflammatory cell infiltration in GRMD. Moreover, treatment with an adeno-associated virus vector encoding a short hairpin RNA (shRNA) that specifically targets NF-*κ*B ameliorated muscle pathologies in *mdx* mice [48]. Therefore, our hDPSC transplantation strategy has the potential to be used in the form of combined therapy with steroid or other immune-modulating treatments.

Comparison of human MSCs derived from different tissues revealed no differences in cell morphology or expression of surface markers typical of mesenchymal stem cells [49]. Many therapeutic approaches have been developed using MSCs derived from bone marrow (BM) [50, 51], adipocytes [52], and placenta [53]. In this study, we first demonstrated the long-term therapeutic effects of systemic administration of DPSCs. hDPSCs showed immunoregulatory properties similar to those of BM-MSCs in terms of the cellular proliferation inhibition of both CD4^+^- and CD8^+^-activated T cells, and increased IL-10 and prostaglandin E2 production compared to BM-MSCs [54]. In addition, hDPSCs proliferate much faster than those from human BM-MSCs, e.g., when the yields of hDPSCs were 1.2 × 10^6^ cells, BM-MSCs were 6.0 × 10^5^ cells at passage 3 [55]. Under serum/xeno-free, good manufacturing practice-compliant (GMP) conditions, DPSCs showed shorter doubling times compared to BM-MSCs and maintained long-term “stemness” [56]. Comparing hDPSCs-cultivation in xeno-free serum and FBS medium, population doublings showed an initial linear trend, but a statistically significantly lower number of cumulative doublings in xeno-free serum versus FBS medium was detected by passage 6 [57]. Based on the safety evaluation of MSC expansion, a consistent decrease in telomere length was found in both DPSC and BM-MSC cultures under GMP conditions [56]. These findings indicate that DPSCs are a promising cell source for transplantation, at least for expansion under the GMP level; however, there is still a need for the development of qualified protocols for clinical-grade expansion of oral MSCs.

While the gene expression profiles of BM-MSCs, adipocyte-MSCs, and umbilical cord tissue-derived MSCs were similar, DPSCs differed in relative pancreatic and duodenal homeobox 1 (PDX1) and Sox2 gene expression and had higher expression of E-cadherin and lower expression of Snail associated with tissue reparative functions in the epithelial-mesenchymal transition [49]. Furthermore, we confirmed that expression of the chemokine, stromal-derived factor-1(SDF-1/CXCL12), from hDPSCs was upregulated in response to TNF-α stimulation (Figure S9). SDF-1 and growth factors might enhance DPSC retention by altering the microenvironment. The binding of SDF-1 to both CXCR4 and CXCR7 is responsible for the production of paracrine mediators, including VEGF, β-FGF-1, and HGF, which exert mitogenic, anti-apoptotic, pro-angiogenic, and anti-inflammatory effects [58]. Comparing to other MSCs, DPSCs have an extensive trophic secretomes, which include NGF, BDNF, NT-3, GDNF, VEGF, and PDGF, and express greater amounts of NGF, BDNF, and NT-3, which promote axon/neurite regeneration [59, 60]. The neuroprotective/pro-regenerative effects are significantly greater in DPSC transplanted animals compared to BM-MSC-treated ones and are correlated with a more favorable neurotrophic secretome by DPSC [60]. Based on this, we surmise that tissue repair mechanisms by DPSC may be associated with DMD treatment. Furthermore, MSCs derived from different sources are transcriptomically different from each other, although they share basic characteristics. This transcriptomic difference is also important in terms of the diversity of the secretions such as mi-RNA. For example, miR-199a-5p is known to be increased in exosomes of DMD patients, or miR-24, which is involved in myogenic differentiation [61]. Future therapeutic studies using various tissue-derived MSCs will allow for the selection of optimal cell sources.

As a possible source for cell therapy, hDPSCs have been investigated for their potential in treating various degenerative diseases such as Alzheimer’s disease, myocardial infarction, bone defects, and corneal reconstruction [62]. In the case of experimental spinal cord injury, stroke, and Parkinson’s disease models, hDPSC transplantation has been demonstrated as a promising treatment for improving functional outcome [63]. Here, we show for the first time the long-lasting restorative effect in DMD animal models produced by systemic DPSC injection that did not result in discernible side effects. Hopefully, this approach can be considered a safe and effective therapeutic measure against DMD. Although further studies are still needed to ascertain the clinical usage and to elucidate molecular mechanisms, since repeated treatments are required to prevent the DMD phenotype, hDPSC treatment can be considered a promising DMD therapy.

## Conclusion

DPSCs have potential as therapeutics, since similar types of bone marrow-derived MSCs have been reported to show immunosuppressive properties. This report investigated the therapeutic effects of DPSCs using animal models of DMD. Our study demonstrates that DMD phenotypes, such as pathological inflammation and motor dysfunction, were significantly improved by repeated systemic injections of DPSCs. This study provides valuable insights into MSC cell therapy in DMD for potential clinical applications.


**Additional file 2: Movie S2.** Movies showing 15 m running analysis in CXMD_J_. Movies of 15 m running in untreated CXMD_J_ littermates (control DMD, 12202MA).**Additional file 3: Movie S3.** Movies showing 15 m running analysis in CXMD_J_. Movies of 15 m running in hDPSC-treated CXMD_J_ (hDPSCs-DMD, 12205MA).

## Supplementary Information

**Additional file 1: Movie S1.** Activity of control and hDPSC-treated CXMD_J_.

**Additional file 4: Figure S1.** Grip strength and daily running distance in aged mice. **Figure S2.** Blood levels of hDPSCs after injection. **Figure S3.** MRI of the lower leg muscle of CXMD_J_. **Figure S4.** H&E staining of hDPSC-treated skeletal muscle. **Figure S5.** Muscle fiber distribution from skeletal muscle of CXMD_J_. **Figure S6.** Clinical follow-up of CXMD_J_ after hDPSC transplantation. **Figure S7.** Multiple parameters of acceleration measured by 15 m of running. **Figure S8.** Reverse transcription PCR of human specific dystrophin expression. **Figure S9.** Cytokine and chemokine expression in hDPSCs. **Table S1.** Normalized grip strength in mice. **Table S2.** Locomotor activity in mice. **Table S3.** Quantitative changes of higher T2-signals in hindlimb muscles.

## Data Availability

The datasets used and/or analyzed during the current study are available from the corresponding author upon reasonable request.

## Abbreviations

ALP: Alkaline phosphatase
AM: Acceleration magnitude
AST: Aspartate transferase
BDNF: Brain-derived neurotrophic factor
BUN: Blood urea nitrogen
BM-MSCs: Bone marrow-derived multipotent mesenchymal stromal cells
BW: Body weight
β-FGF-1: β-Fibroblast growth factor-1
CD: Cluster of differentiation
CNFs: Centrally nucleated fibers
CRP: C-reactive protein
CXCR: C-X-C Chemokine receptor
CXMD_J_: Canine X-linked muscular dystrophy model in Japan
dMyHC: Developmental myosin heavy chain
DAPI: 4, 6-Diamidino-2-phenylindole
DMD: Duchenne muscular dystrophy
DPSCs: Dental pulp stem cells
EDL: Extensor digitorum longus
EF: Ejection fraction
ELISA: Enzyme-linked immunosorbent assay
FHL: Flexor hallucis longus
GDNF: Glial Cell Line-derived Neurotrophic Factor
GL: Gastrocnemius lateral
GM: Gastrocnemius medialis
GRMD: Golden retriever muscular dystrophy
H&E: Hematoxylin and eosin
HGF: Hepatocyte growth factor
HLA-DR: Human leukocyte antigen-D-related
IL-6: Interleukin-6
IL-10: Interleukin-10
LV: Left ventricular
MRI: Magnetic resonance imaging
MSCs: Multipotent mesenchymal stromal cells
N.D.: Not detected
NF-κB: Nuclear factor-kappa B
NGF: Nerve growth factor
NT-3: Neurotrophin-3
PCR: Polymerase chain reaction
PDGF: Platelet-Derived Growth Factor
PDX1: Pancreatic and duodenal homeobox 1
ROI: Regions of interest
RV: Right ventricular
shRNA: Short hairpin RNA
SD: Standard deviation
SDF-1/CXCL12: stromal-derived factor-1
SNRs: Signal-to-noise ratios
Sox2: Sry related high mobility group box 2
TA: Tibialis anterior
TNF-α: Tumor necrosis factor-alpha
VEGF: Vascular endothelial growth factor
WT: Wild type
