# Supplementary material for: Dental pulp stem cells can improve muscle dysfunction in animal models of Duchenne muscular dystrophy
Source: Stem Cell Res Ther. 2021 Jan 25;12:78. doi: 10.1186/s13287-020-02099-3 (PMC7831244; doi:10.1186/s13287-020-02099-3)
Supplement: Supplementary file 4 — Additional file 4: Figure S1. Grip strength and daily running distance in aged mice. Figure S2. Blood levels of hDPSCs after injection. Figure S3. MRI of the lower leg muscle of CXMDJ. Figure S4. H&E staining of hDPSC-treated skeletal muscle. Figure S5. Muscle fiber distribution from skeletal muscle of CXMDJ. Figure S6. Clinical follow-up of CXMDJ after hDPSC transplantation. Figure S7. Multiple parameters of acceleration measured by 15 m of running. Figure S8. Reverse transcription PCR of human specific dystrophin expression. Figure S9. Cytokine and chemokine expression in hDPSCs. Table S1. Normalized grip strength in mice. Table S2. Locomotor activity in mice. Table S3. Quantitative changes of higher T2-signals in hindlimb muscles. [file 13287_2020_2099_MOESM4_ESM.zip › Fig S (Kasahara) 201128.pdf]

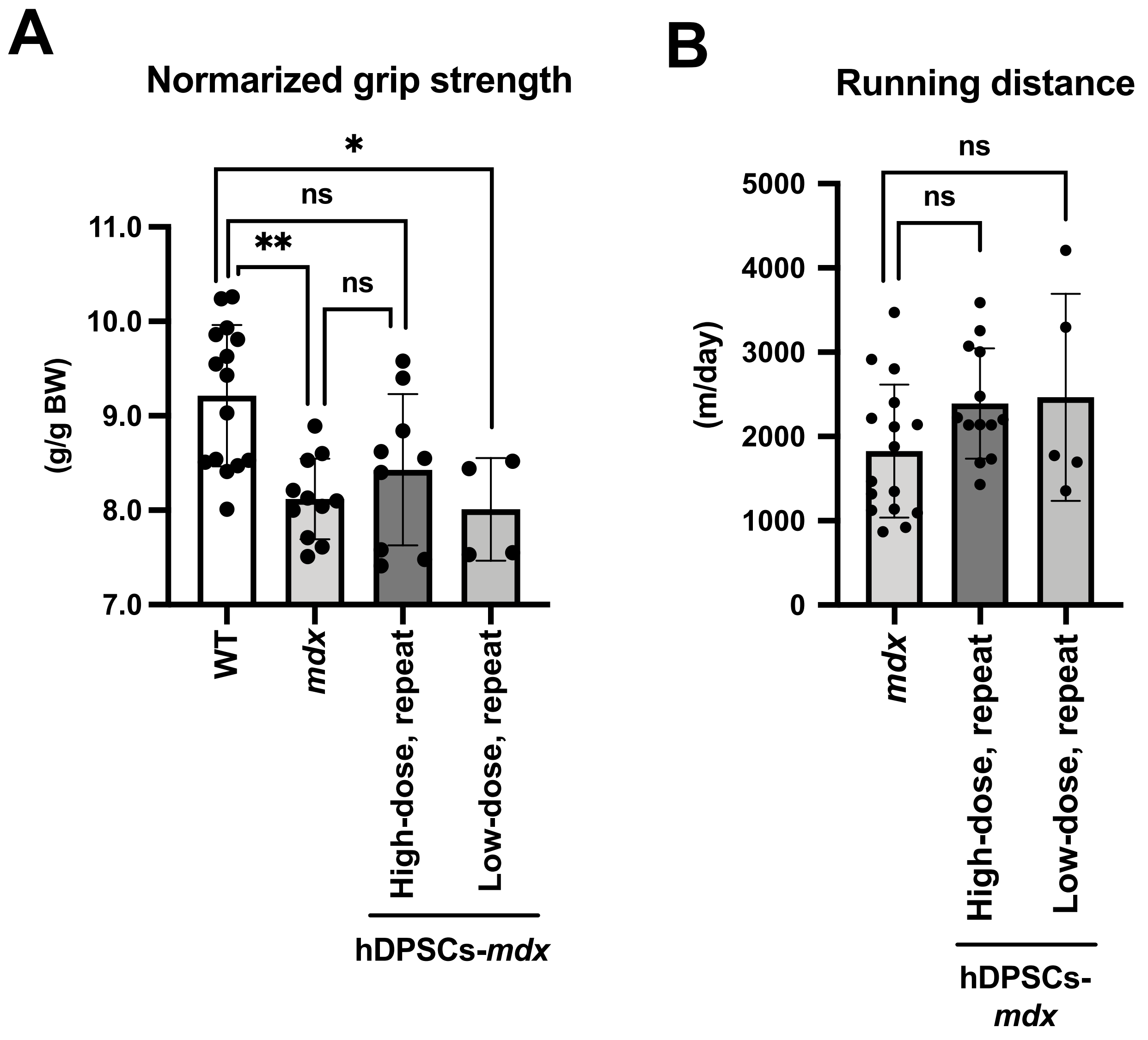

Supplemental Figure 1

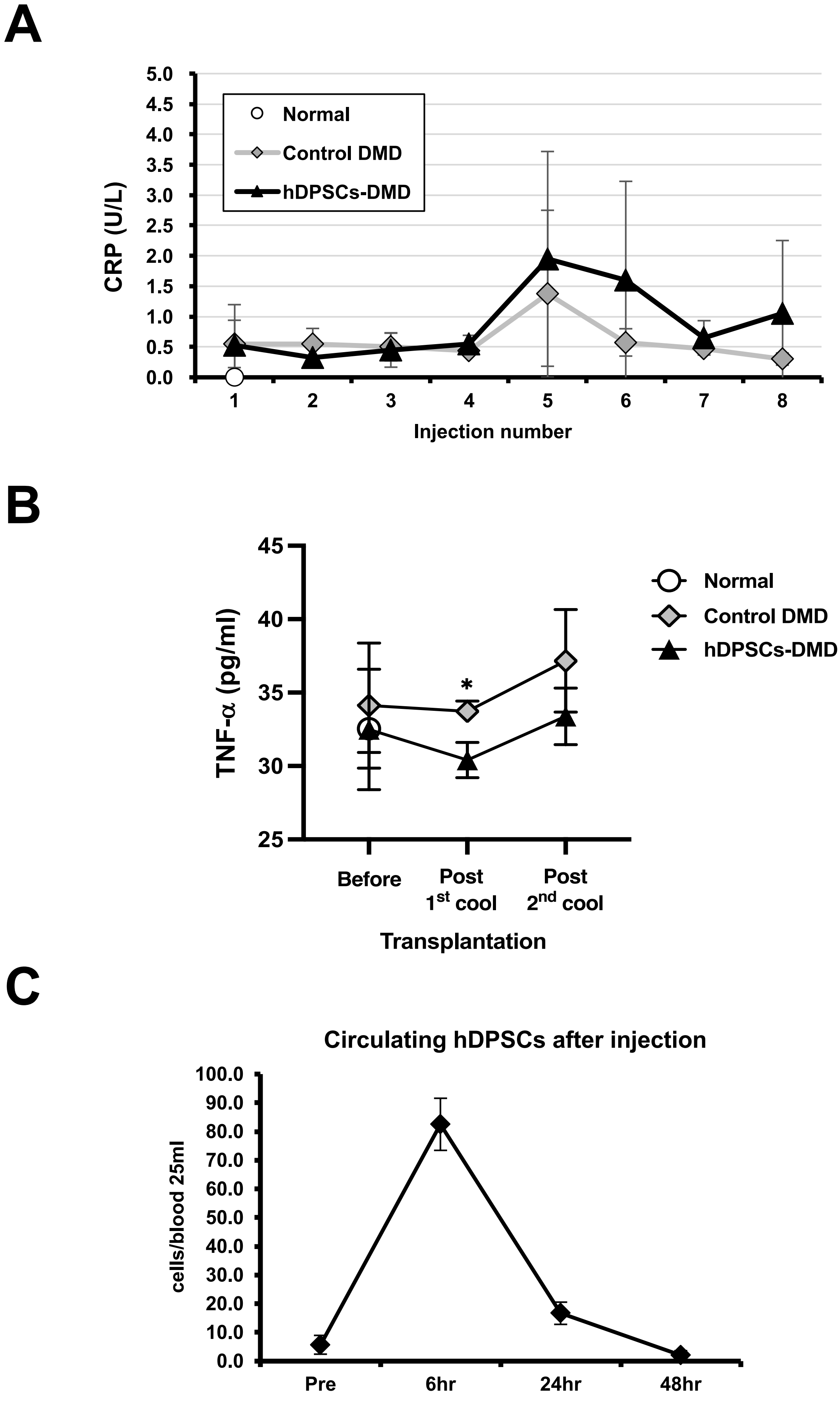

Supplemental Figure 2

Control DMD

12 weeks old
12202MA

hDPSCs-DMD

Post-2nd cool injection
12205MA

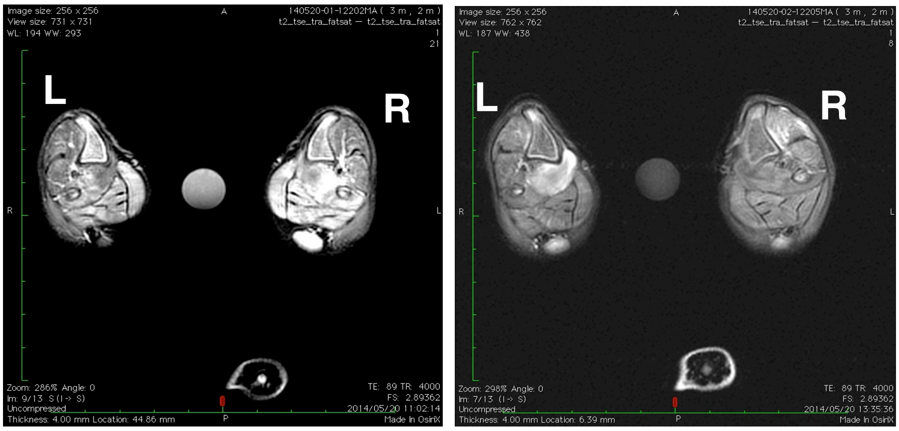

Supplemental Figure 3

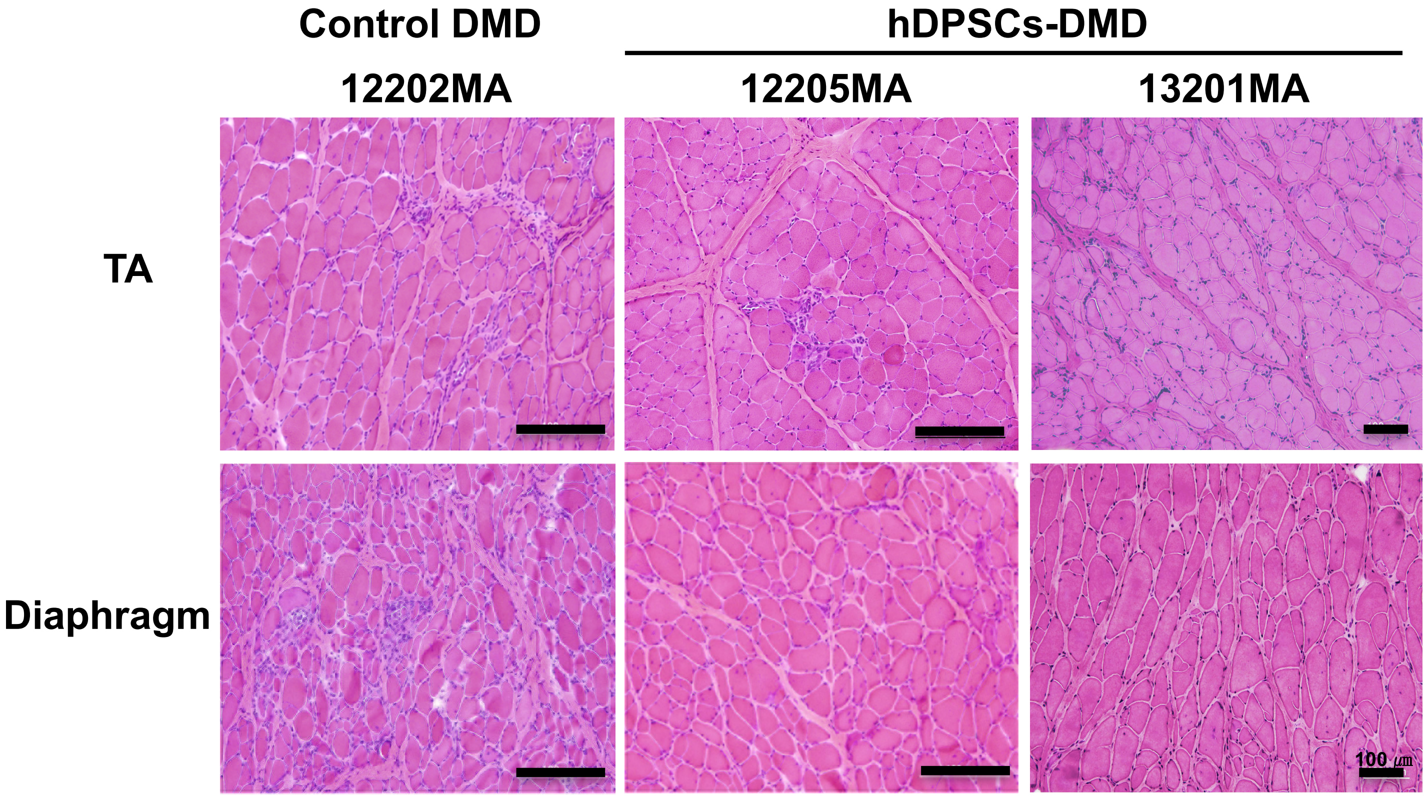

Supplemental Figure 4

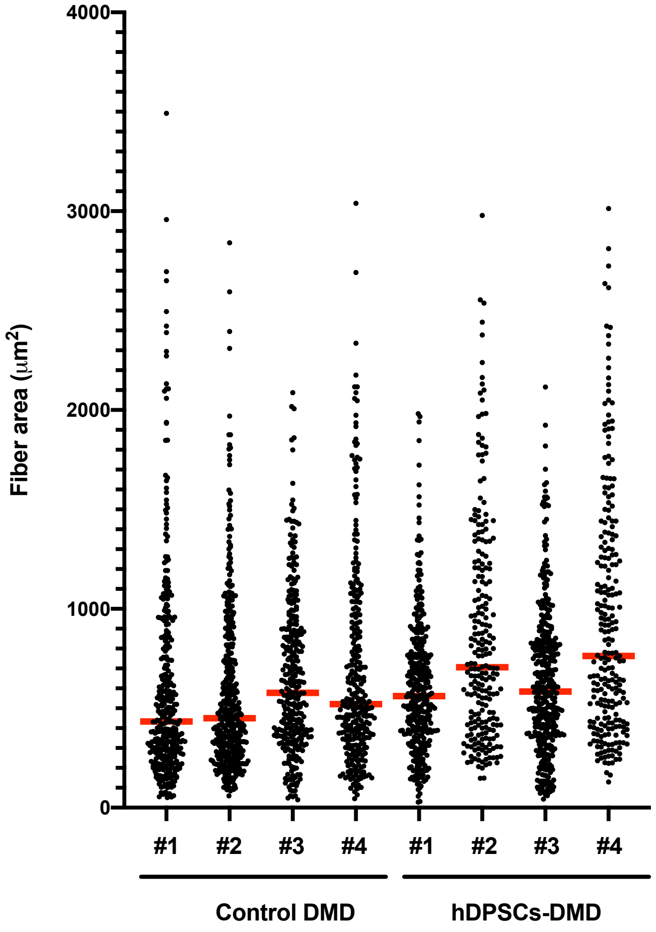

Supplemental Figure 5

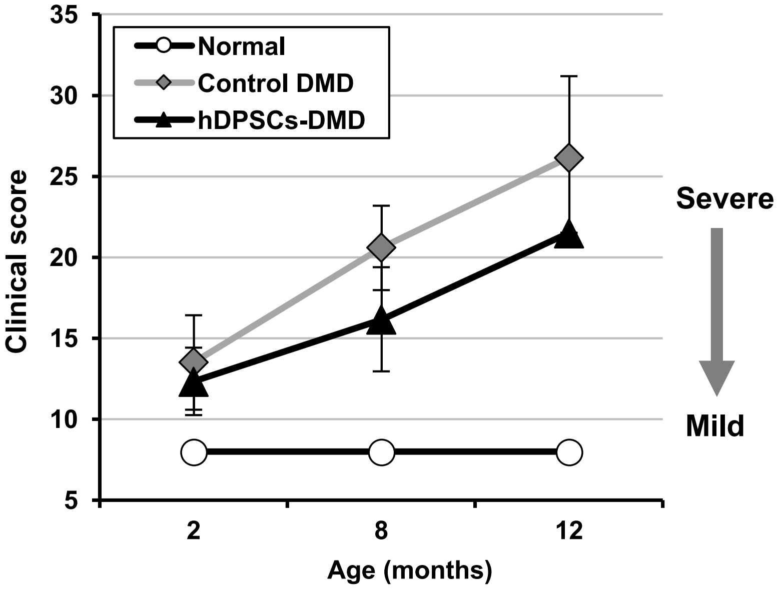

Supplemental Figure 6

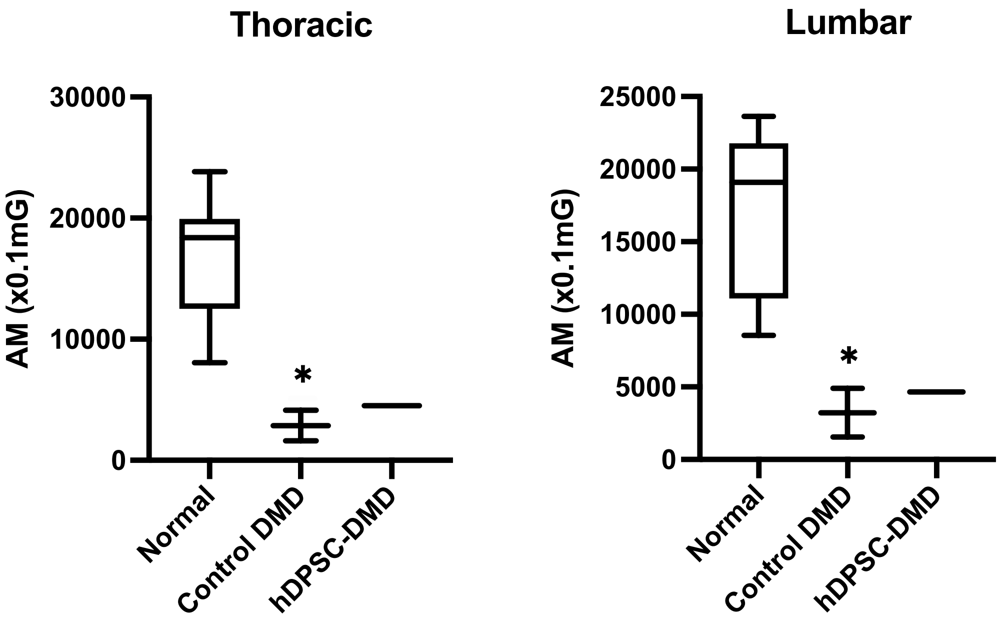

Supplemental Figure 7

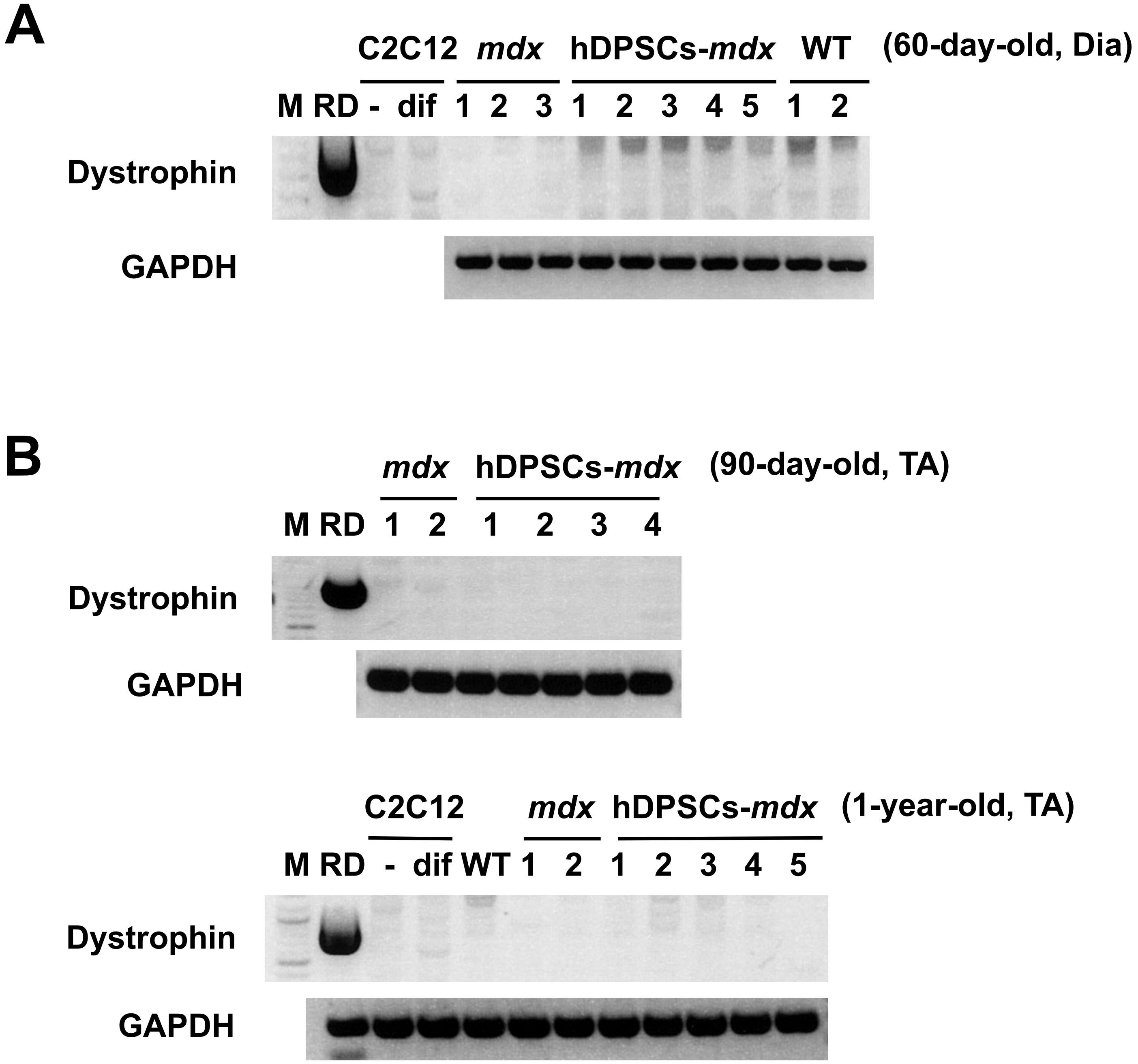

Supplementary Figure 8

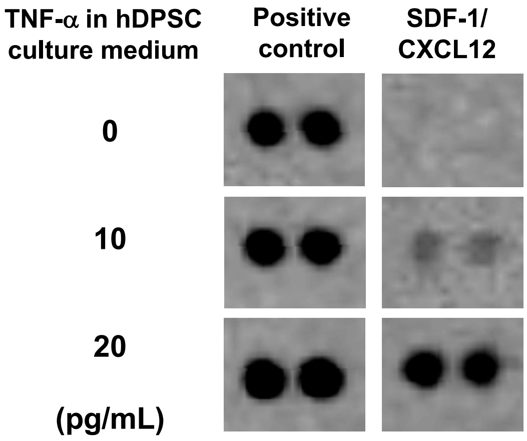

Supplemental Figure 9
