# Supplementary material for: Dental pulp stem cells can improve muscle dysfunction in animal models of Duchenne muscular dystrophy
Source: Stem Cell Res Ther. 2021 Jan 25;12:78. doi: 10.1186/s13287-020-02099-3 (PMC7831244; doi:10.1186/s13287-020-02099-3)
Supplement: Supplementary file 4 — Additional file 4: Figure S1. Grip strength and daily running distance in aged mice. Figure S2. Blood levels of hDPSCs after injection. Figure S3. MRI of the lower leg muscle of CXMDJ. Figure S4. H&E staining of hDPSC-treated skeletal muscle. Figure S5. Muscle fiber distribution from skeletal muscle of CXMDJ. Figure S6. Clinical follow-up of CXMDJ after hDPSC transplantation. Figure S7. Multiple parameters of acceleration measured by 15 m of running. Figure S8. Reverse transcription PCR of human specific dystrophin expression. Figure S9. Cytokine and chemokine expression in hDPSCs. Table S1. Normalized grip strength in mice. Table S2. Locomotor activity in mice. Table S3. Quantitative changes of higher T2-signals in hindlimb muscles. [file 13287_2020_2099_MOESM4_ESM.zip › Supplementary_information_SCRT-D-20-01153 201126.docx]

**Supplementary Information**

**Figure S1. Grip strength and daily running distance in aged mice**

(**A**) Normalized grip strength (g/g BW) measured in 50–58-week-old C57BL/6 (WT, n = 15), untreated *mdx* (n = 11)*,* and repeat hDPSC (high- or low-dose; n = 9, 4)-treated *mdx* mice. (**B**) Quantitated daily running distance covered during wheel running in 50-week-old untreated *mdx* (n = 17)*,* and repeat hDPSC (high- or low-dose; n = 13 or 5)-treated *mdx* mice. The data were determined from a period of 5 days for each mouse. All data are represented as mean ± SD and statistical differences compared to WT (^*^*P* < 0.05, ^**^*P* < 0.01). WT: wild type; BW: body weight; hDPSCs: human dental pulp stem cells

**Figure S2. Blood levels of hDPSCs after injection**

(**A**) Serum chemistry data of normal, untreated (control DMD; 12202MA, 13303MA, 14102MA), and hDPSC-treated CXMD_J_ (hDPSCs-DMD; 12205MA, 13201MA, 13304MA) dogs after each administration throughout the experimental period. CRP levels were measured using a colorimetric assay with a FDC3500 clinical biochemistry analyzer. (**B**) Quantitative serum levels of TNF-α in the normal, untreated (control DMD; 13303MA) and hDPSC-treated CXMD_J_ (hDPSCs-DMD; 13304MA) dogs at before (1–2-month-old), post 1^st^ cool (3–4-months-old)-, and 2^nd^ cool transplantation (7–8-month-old) was determined using ELISA. n = 3, *t*-test. Data are represented as mean ± SD and statistical differences compared to control DMD (^*^*P* < 0.05). (**C**) Relative amounts of hDPSCs in blood were determined using human-specific *Alu-*based quantitative real-time PCR (qPCR). To quantify the amount of human DNA from hDPSCs, qPCR was performed using genomic DNA prepared from total blood (25 μL) of a hDPSC-treated CXMD_J_ (12205MA) at 0, 6, 24, and 48 h post-injection. hDPSCs: human dental pulp stem cells; CXMD_J_: canine X-linked muscular dystrophy model in Japan; DMD: Duchenne muscular dystrophy; CRP: C-reactive protein

**Figure S3. MRI of the lower leg muscle of CXMD_J_**

Cross-sectional MRI of the lower leg muscles of untreated CXMD_J_ and hDPSC-treated CXMD_J_ (control DMD, 12202MA; hDPSCs-DMD, 12205MA). Muscle necrosis and inflammation based on a sequence of T2-weighted imaging were comparable in the lower legs (R, right side; L, left side, left/right asymmetry) of CXMD_J_ at 12 weeks of age, and in littermates. CXMD_J_: canine X-linked muscular dystrophy model in Japan; MRI: magnetic resonance imaging; DMD: Duchenne muscular dystrophy; hDPSCs: human dental pulp stem cells

**Figure S4. H&E staining of hDPSC-treated skeletal muscle**

H&E staining of the TA muscles and diaphragm from the untreated (control DMD, 12202MA) and hDPSC-treated CXMD_J_ (hDPSCs-DMD, 12205MA, 13201MA). Scale bars, 100 μm. H&E: hematoxylin and eosin; hDPSCs: human dental pulp stem cells; TA: tibialis anterior; CXMD_J_: canine X-linked muscular dystrophy model in Japan; DMD: Duchenne muscular dystrophy

**Figure S5. Muscle fiber distribution from skeletal muscle of CXMD_J_**

Muscle fiber areas (μm^2^) measured from the TA muscles of control- and hDPSC-DMD dogs by H&E staining (n = 4, #1−4). Each fiber area is shown as a dot, and the average fiber area is described as a red bar in each muscle. H&E: hematoxylin and eosin; hDPSCs: human dental pulp stem cells; TA: tibialis anterior; CXMD_J_: canine X-linked muscular dystrophy model in Japan; DMD: Duchenne muscular dystrophy

**Figure S6. Clinical follow-up of CXMD_J_ after hDPSC transplantation**

For clinical evaluation, a comprehensive analysis was performed for gait and mobility disturbance, limb muscle atrophy, temporal muscle atrophy, drooling, macroglossia, dysphagia, and abnormal sitting posture [1, 2]. The severity of each sign was classified using a score from 1 to 5 for CXMD_J_ (grade 1, none; grade 5, severe). The total score of the clinical grading exam was calculated at the ages of 2 to 12 months while comparing normal dogs and CXMD_J_ littermates (control DMD and hDPSCs-DMD). hDPSCs: human dental pulp stem cells; CXMD_J_: canine X-linked muscular dystrophy model in Japan; DMD: Duchenne muscular dystrophy

**Figure S7. Multiple parameters of acceleration measured by 15 m of running**

To determine the acceleration parameter, we used portable wireless hybrid sensors (TSND121; ATR-Promotions) on the thoracic and lumbar regions of dogs, as previously described [3]. The acceleration magnitude (*AM*) was calculated from the three acceleration vectors (*Ax*, *Ay*, *Az*) as the square root of the sum of the three axial values (*AM* = √*Ax*^2^ + *Ay*^2^ + *Az*^2^) [4], and was averaged for each trial. The relative components of the *AM* along the three axes (%) were calculated by dividing the absolute values of each axis by the *AM* [5], and these components that were averaged in each trial were calculated as acceleration ratios (*Ax* ratio, *AY* ratio, *AZ* ratio). The *AM* value was calculated from the thoracic and lumbar regions in normal dogs (13301MN and 14103MN), untreated littermates (control DMD, 13303MA, 14102MA), and hDPSC-treated CXMD_J_ (hDPSC-DMD, 13304MA). All data are represented as mean ± SD and statistical differences compared to normal (^*^*P* < 0.05). CXMD_J_: canine X-linked muscular dystrophy model in Japan; DMD: Duchenne muscular dystrophy

**Figure S8. Reverse transcription PCR of human specific dystrophin expression**

To determine dystrophin expression derived from hDPSCs in the skeletal muscle, RNA was isolated from the diaphragm (**A**) and TA muscles (**B**) of 60-, 90-day-, or 1-year-old WT mice, *mdx* mice (#1–3 or #1–2), repeated high-dose of hDPSC-treated *mdx* mice (#1–5 or #1–4), human rhabdomysarcoma (RD) cells, mouse myoblast cells (C2C12) as a control, undifferentiated (-), or differentiated cells (dif) under 2% horse serum culture. Reverse transcription PCR was performed using human-specific dystrophin and GAPDH an internal control. The primers used in this study were as follows: human dystrophin forward, 5- TGAAACTGGAGGACCCGTG-3 (1^st^ reaction), 5- AAAAGACCTTGGGCAGCTTG-3 (2^nd^ reaction) and reverse, 5- CCAAGAGGCATTGATATTCTC-3; and canine dystrophin: forward, GAACGAGCCCCTTCCTTT-3′; and reverse, 5- CATTTGTTCCTGCACTTCG-3′. As an internal control, a primer set for the housekeeping gene GAPDH was used as follows: canine, forward, 5-GCGAGATCCCGCCAACATCAAA-3, and reverse, 5- AGGAGCAGAGATGATGACCCTC-3′. PCR products were subjected to agarose-gel electrophoresis. M: marker; TA: tibialis anterior; WT: wild type; hDPSCs: human dental pulp stem cells; CXMD_J_: canine X-linked muscular dystrophy model in Japan; DMD: Duchenne muscular dystrophy; GAPDH: glyceraldehyde-3-phosphate dehydrogenase

**Figure S9. Cytokine and chemokine expression of hDPSCs**

hDPSCs were cultured for 2 days in serum-free medium with or without TNF-α (10, 20 pg/mL) for array analysis. Cytokine and chemokine expression in the medium was analyzed using the Proteome ProfilerTM Array. Changes in the expression levels of stromal-derived factor-1 (SDF-1/CXCL12), compared to positive control signals, in response to TNF-α stimulation were quantified using Image Quant LAS 4000 coupled with Image Quant TL software (GE Healthcare). Array images are presented. hDPSCs: human dental pulp stem cells

**Table S1. Normalized grip strength in mice**

|  |  | WT | *mdx* | High-dose hDPSC-treatment | |  | Low-dose hDPSC-treatment | |
| --- | --- | --- | --- | --- | --- | --- | --- | --- |
|  |  |  |  | Repeat | Single |  | Repeat | Single |
| Young  (9 weeks old) | Actual mean  (g/ g BW) | 9.69 | 7.14 | 10.06 | 8.31 |  | 7.93 | 8.92 |
|  | SD of discrepancy | 0.49 | 2.31 | 1.11 | 0.34 |  | 1.90 | 1.01 |
|  | Number of values | 6 | 9 | 17 | 3 |  | 7 | 7 |
| Aged  (50 weeks old) | Actual mean  (g/ g BW) | 9.21 | 8.12 | 8.43 |  |  | 8.01 |  |
|  | SD of discrepancy | 0.75 | 0.43 | 0.80 | - |  | 0.54 | - |
|  | Number of values | 15 | 11 | 9 |  |  | 4 |  |

Normalized grip strength (g/g BW) measured in 9-week-old (young) and 50-week-old (aged) WT, untreated *mdx,* repeat, and single hDPSC (high- or low-dose)-treated *mdx* mice. BW: body weight; WT: wild type; hDPSC: human dental pulp stem cells

**Table S2. Locomotor activity in mice**

|  |  |  | WT | *mdx* | High-dose hDPSC-treatment | |  | Low-dose hDPSC-treatment | |
| --- | --- | --- | --- | --- | --- | --- | --- | --- | --- |
|  |  |  |  |  | Repeat | Single |  | Repeat | Single |
| Maximum running speed (m/min) | Young (12 weeks old) | Actual mean | 28.7 | 18.71 | 25.6 | 22.74 |  | 25.76 | 25.11 |
|  |  | SD of discrepancy | 4.49 | 4.31 | 2.81 | 2.46 |  | 5.16 | 4.48 |
|  |  | Number of values | 8 | 12 | 9 | 3 |  | 6 | 6 |
|  | Aged (50 weeks old) | Actual mean | 20.4 | 17.43 | 20.61 | - |  | 19.35 | - |
|  |  | SD of discrepancy | 2.46 | 3.06 | 1.602 |  |  | 5.329 |  |
|  |  | Number of values | 3 | 19 | 14 |  |  | 5 |  |
| Dairy running distance (m/day) | Young (12 weeks old) | Actual mean | 4305.0 | 1976.0 | 3353.0 | 3415.0 |  | 3820.0 | 3357.0 |
|  |  | SD of discrepancy | 966.1 | 456.0 | 493.7 | 448.3 |  | 818.3 | 1190.0 |
|  |  | Number of values | 6 | 10 | 8 | 3 |  | 5 | 6 |
|  | Aged (50 weeks old) | Actual mean | 2854.0 | 1827.0 | 2392.0 | - |  | 2466.0 | - |
|  |  | SD of discrepancy | 609.2 | 789.9 | 654.6 |  |  | 1229.0 |  |
|  |  | Number of values | 5 | 16 | 13 |  |  | 5 |  |

Quantification of maximum running speed (m/min) in the wheel cage, and daily distance covered during wheel running in 12-week-old (young) and 50-week-old (aged) WT, untreated *mdx,* repeat, and single hDPSC (high- or low-dose)-treated *mdx* mice. WT: wild type; hDPSC: human dental pulp stem cells

**Table S3. Quantitative changes of higher T2-signals in hindlimb muscles**

| Age | 2 months | | 7 months | | Relative value | |
| --- | --- | --- | --- | --- | --- | --- |
| Muscle of control DMD (Dog ID) | SNR | √SD2ROI-(Sdnoise/0.665)^2^ | SNR | √SD2ROI-(Sdnoise/0.665)^2^ | SNR | √SD2ROI-(Sdnoise/0.665)^2^ |
| L-GM (13303MA) | 139.5 | 57.2 | 93.0 | 35.3 | 0.67 | 0.62 |
| L-GL (13303MA) | 123.8 | 48.8 | 85.2 | 27.2 | 0.69 | 0.56 |
| L-GL (14102MA) | 127.0 | 80.7 | 82.0 | 56.7 | 0.65 | 0.70 |
| L-GL (14102MA) | 129.7 | 47.3 | 86.1 | 37.2 | 0.66 | 0.79 |
| L-GL (13102MA) | 147.9 | 80.5 | 119.3 | 39.4 | 0.81 | 0.49 |
| L-FDS (13102MA) | 129.6 | 80.0 | 113.8 | 51.9 | 0.88 | 0.65 |

| Age | 2 months | | 7 months | | Relative value | |
| --- | --- | --- | --- | --- | --- | --- |
| Muscle of hDPSC- DMD (Dog ID) | SNR | √SD2ROI-(Sdnoise/0.665)^2^ | SNR | √SD2ROI-(Sdnoise/0.665)^2^ | SNR | √SD2ROI-(Sdnoise/0.665)^2^ |
| R-GL (13202MA) | 180.5 | 31.5 | 95.3 | 36.1 | 0.53 | 0.53 |
| L-GM (13202MA) | 191.6 | 31.4 | 117.0 | 39.2 | 0.61 | 0.61 |
| R-FHL (13304MA) | 123.8 | 107.9 | 71.3 | 29.9 | 0.58 | 0.58 |
| L-EDL (13304MA) | 122.6 | 64.6 | 77.7 | 43.4 | 0.63 | 0.63 |

Cross-sectional magnetic resonance imaging (MRI) of the lower leg muscles of canines was performed. Quantitative changes of higher T2-signals (signal-to-noise ratio, SNR) in the hindlimb muscles on CXMD_J_ are shown in the MRI data. Relative SNR was calculated from the highest signals in each hindlimb of 2-month-old dogs compared to 7-month-old dogs. Data are represented as mean ± SD and statistical differences compared to control-DMD (^*^*P* < 0.05, *t*-test). DMD: Duchenne muscular dystrophy; CXMD_J_: canine X-linked muscular dystrophy model in Japan

**Movie S1. Activity of control and hDPSC-treated CXMD_J_**

**Movie S2. Movies showing 15 m running analysis in CXMD_J_**

**Movies of 15 m running in untreated CXMD_J_ littermates (control DMD, 12202MA)**

**Movie S3. Movies showing 15 m running analysis in CXMD_J_**

**Movies of 15 m running in the hDPSC-treated CXMD_J_ (hDPSCs-DMD, 12205MA).**

**References**

1. Shimatsu Y, Katagiri K, Furuta T, Nakura M, Tanioka Y, Yuasa K, Tomohiro M, Kornegay JN, Nonaka I, and Takeda S: **Canine X-linked muscular dystrophy in Japan (CXMD_J_).** *Exp Anim.* 2003, **52:** 93-97.

2. Hayashita-Kinoh H, Yugeta N, Okada H, Nitahara-Kasahara Y, Chiyo T, Okada T, and Takeda S: **Intra-amniotic rAAV-mediated microdystrophin gene transfer improves canine X-linked muscular dystrophy and may induce immune tolerance.** *Mol Ther.* 2015, **23:** 627-637.

3. Kuraoka M, Nitahara-Kasahara Y, Tachimori H, Kato N, Shibasaki H, Shin A, Aoki Y, Kimura E, and Takeda S: **Accelerometric outcomes of motor function related to clinical evaluations and muscle involvement in dystrophic dogs.** *PLoS One.* 2018, **13:** e0208415.

4. Galan-Mercant A, Baron-Lopez FJ, Labajos-Manzanares MT, and Cuesta-Vargas AI: **Reliability and criterion-related validity with a smartphone used in timed-up-and-go test.** *Biomed Eng Online.* 2014, **13:** 156.

5. Barthelemy I, Barrey E, Aguilar P, Uriarte A, Le Chevoir M, Thibaud JL, Voit T, Blot S, and Hogrel JY: **Longitudinal ambulatory measurements of gait abnormality in dystrophin-deficient dogs.** *BMC Musculoskelet Disord.* 2011, **12:** 75.
